# “I can’t live like that”: the experience of caregiver stress of caring for a relative with substance use disorder

**DOI:** 10.1186/s13011-021-00344-3

**Published:** 2021-01-15

**Authors:** Geoffrey Maina, Marcella Ogenchuk, Taryn Phaneuf, Abukari Kwame

**Affiliations:** 1 College of Nursing, University of Saskatchewan, Prince Albert Campus, 173 130- 1061 Central Avenue, Prince Albert, S6V 4W4 Canada; 2 College of Nursing, University of Saskatchewan, 104 Clinic Place, HSc E Wing, 4226, Saskatoon, SK S7N 5E5 Canada; 3 University of Saskatchewan, Saskatoon, SK S7N 5E5 Canada

**Keywords:** Substance use, Caregiver distress, Self-care

## Abstract

**Background:**

The impact of addiction extends beyond the individual using a substance. Caring for an individual with addiction creates persistent stressful circumstances that cause worry, anger, depression, shame, guilt, anxiety, and behavioral problems within the family unit.

**The aim of the study:**

The paper aims to explore the experiences of caring for a relative with a substance use disorder (SUD) and self-care strategies caregivers employ.

**Methods:**

The study adopted an exploratory qualitative design. To be included in the study, participants were required to have a relative with a (SUD) disorder and not be actively using the substance themselves. Individual interviews were conducted to gather their experiences, meanings, and how they made sense of caring for a relative with a SUD.

**Results:**

Twenty one participants were involved in the study, of which 17 were women, and four were men of which there had a sister, four had a brother, eight had a parent, six had a dependent, and one participant had a grandparent with a SUD. Four themes, whose overarching focus is the pains of living and caring for a family with a SUD, caused the participants and how the participants mitigated these experiences

**Conclusion:**

The stress associated with caring for individuals with a SUD impacts the caregiver’s physical and mental health. Specific care modalities targeting caregivers need to be developed to address the health impact and to support self-care.

## Introduction

Addiction to substances arises from a long-term and repeated misuse of high doses of substances that impede an individual’s health and quality of life [1]. The impact of substance use extends beyond the individual using a substance to include family and community members [2]. Substance use impacts a family’s everyday life, health, social relations, family relationships, roles, rituals, routines, and careers, as well as finances [3, 4]. In Canada, it is estimated that 660,000 children under the age of 18 live in households with at least one alcoholic parent. Although many children of parents with a Substance use disorder (SUD) do not experience neglect or abuse, they are at an increased risk for child maltreatment and different forms of abuse, which can contribute to psychological damage, and make them more vulnerable to their own substance use [1, 5, 6].

Although much is known about the impact of substance use to families, treatments and programs are often devoted to treating addiction and do not often include the entire family members in the recovery process. With the growing substance use crisis in the community, the need to understand how it affects families was expressed during a community engagement and knowledge exchange event where participants identified the lack of support for families affected by substance use as a significant gap in care. The paper aims to explore the psychological impact of caring for a relative with a SUD and how these families engage in self-care. This manuscript is a result of a larger study set in a midsized city in a prairie province, where rampant substance use profoundly impacts the community, including early exposure to children and youth [7, 8]. Three research questions guide this study;

- What are the experiences of caring for a relative with a SUD?
- What factors shape these experiences?
- What self-care strategies do families affected by substance use to promote self-care?

### Brief literature review

Substance use and addiction impact families in different ways. Children exposed to illicit substances are at risk of developing psychiatric issues such as anxiety or depression, behavioral and cognitive difficulties that can affect their learning abilities [9–11]. Moreover, children of parents with substance use are at a higher risk of experiencing maltreatment and other negative outcomes that are commonly referred to as adverse childhood experiences such as abuse and neglect and are hence more likely to be placed in foster homes and develop their addiction later in life [12].

Substance use can lead to marital disintegration, violence, conflict, child neglect, and legal issues [13, 14]. Families living with a close member with substance use often experience unmet needs, impaired attachments, economic hardships, emotional distress, and can even experience violence [2]. Moreover, caring for a partner or a dependent with a SUD creates persistent stressful circumstances that can cause worry, anger, depression, shame, guilt, anxiety, and behavioral problems within the family [13, 15]. The stress and crisis associated with caring for a relative with a SUD can impede the family’s functionality [16].

Chronic stress can cause an individual to adopt maladaptive behaviors and offset the body’s natural balance, and have harmful physical effects [2, 17, 18]. Mental health impacts such as depression and anxiety have been reported among relatives with a substance use disorder [19, 20]. In addition, having a close relative with substance use also creates stressful life circumstances that can increase family member’s risk of experience strain that can lead to physical and physiological ill-health [21].

Considering the impact of substance use on relatives, it is therefore essential that when examining the impact of addiction on families, attention be paid to how it affects each member of the family [22]. Self-care practices for families affected by addiction may include attending a personal counseling session, immersion in the individual’s care and recovery, self-directed education on addiction, family treatment involvement, and finding support [14]. Social support is essential during stressful experiences, as positive support can reduce stress and its side effects while increasing resilience [23].

## Methods

The study adopted an exploratory qualitative design to explore families’ experiences affected by addiction and to understand the resources needed to support them. A qualitative study design was proposed because it is an ideal methodology for exploring a topic. We used purposeful sampling to recruit participants by placing posters in strategic locations such as the library and public notice boards through word-of-mouth and email dissemination. Participants were required to have a relative with a SUD such as parents, siblings, children, partners, or grandparents to be included in the study. We aimed for 21 participants to have a variety of participation based on the relationship with the relative with a substance use disorder. Preceding the study’s commencement, we obtained the participants’ verbal and written consent and the University of Saskatchewan’s Ethics Board approval.

A semi-structured interview guide was used to guide the interview, which was conducted by GM, a researcher with experience and expertise in qualitative research methods. The interviews focused on the participants’ experiences of growing up, their experiences with substance use, their perceptions of the impact of substance use on them and their families, and the coping strategies they employed. Each interview lasted for an average of 28 min and was recorded with a digital voice recorder. Verbatim transcription ensued. Professional counseling was made available to participants who wished to debrief after the interview.

The research team included researchers, community members with lived experiences, and health care managers who came together to map the data exploration that led to the initial coding framework development. Two research assistants independently analyzed the interviews using NVIVO 12, a qualitative research data management software system.

## Results

Twenty-one individuals participated in the study and included four males and 17 females whose ages ranged from 27 to 72. The details of relatives with a SUD are summarised in Table 1. Overall, 13 of the participants grew up in homes where relatives used the substance at an early age. Four themes, whose overarching focus is on the psychological impact of caring for a relative with a SUD, are discussed: 1) grieving the loss, 2) living in dread and despair, 3) living in perpetual crisis, and 4) mitigating the impact of substance use in the family.

**Table 1:** Participant’s information of relatives with a SUD

Participant	Age	Gender	Person with Addiction
PA.001	44	Female	Sister addicted to opiated for more than 5 years
PA.002	56	Female	Brother is addicted to alcohol and a history of cocaine use.
PA.003	48	Female	Dependents are addicted to methamphetamine.
PA.004	57	Female	Daughter who is addicted to methamphetamine.
PA.005	57	Female	Children are addicted to substances- methamphetamine. Cares for her grandchildren because the daughter is unable to.
PA.006	28	Male	Sisters and father are addicted to drugs and alcohol. One sister has HIV, Hep C, TB, and diabetes.
PA.007	27	Female	Sister is addicted to methamphetamine for 13 years; brother struggles with alcohol, and the mother is addicted to opiates and gambling.
PA.008	50	Female	Two brothers are addicted to alcohol and drugs and have Hepatitis C. Ex-husband was addicted to alcohol.
PA.009	37	Female	Father struggles with alcohol addiction.
PA.010	50	Male	Ex-wife’s father struggles with alcohol addiction
PA.011	54	Male	Two sons are addicted to alcohol and drugs and also suffer from HIV.
PA.012	45	Female	Stepfather is addicted to alcohol
PA.013	63	Female	Son is addicted to alcohol and methamphetamine.
PA.014	37	Female	Father is addicted to alcohol
PA.015	38	Female	Brother is addicted to alcohol and used cocaine and methamphetamine.
PA.016		Female	Father is addicted to alcohol, and her mother died from a methadone overdose.
PA.017	72	Male	Daughter is addicted to alcohol; grandson, 22 struggles with alcohol addiction.
PA.018		Male	Father was addicted to cocaine for more than 10 years.
PA.019	22	Female	Brother is addicted to alcohol and cocaine.
PA.020	62	Female	Daughter and boyfriend are addicted to drugs
PA.021	35	Female	Three sisters are addicted to alcohol

### Theme 1: grieving the loss

The loss of the relationship between the family and the relative with a SUD was frequently mentioned as a significant consequence of impact addiction. SUD’s relative is often unable to maintain the relationship dynamics needed to support sound and healthy co-existence with all members. For many participants, substance use robbed them of a relationship with the person using substances, which was akin to losing their relative. Even then, relatives continued to expend their time, energy, and money into caring for the relative affected by SUD to keep them safe or alive, with the hope that the lost relationship would be restored. After endless efforts did not yield the desired fruit, some participants signaled their loss of hope for recovery and actively grieved the loss of the relationship and the person:

*I know it sounds cold, washed my hands of him because I have given so much to try and help him. So much of my life that there's nothing else I feel that I can do. I can't put more time and effort into helping him if he doesn't want to help himself. I've lost a brother because of drugs and substance use* (38 years, sister).

Grieving the loss of a relationship was sometimes accompanied by an intentional decision to sever the relationship with the relative affected by SUD. Yet, the diminished connection did not permit the caregiver to move on with their life. Instead, it complicated the grief process:

*It impacted me a lot. I've had a lot of grief. I felt like I did lose my son [light sobbing], I lost the son I had, now there is this new man with this illness.* (63 years, mother).

The severity of the loss relationship in this quote was significant since SUD’s relative was perceived as a different person and referred to as ‘this new man.’ Participants also grieved the consequences of living with an individual with a SUD. It was not uncommon for the participants to report a material loss when they allowed the individual with SUD to cohabit with them. The company, the individual, kept also worried some participants. Living in such an environment took away the joy of home and, although participants were not living on the street, they felt unease due to the regular conflict and apprehension. Subsequently, they felt as though they had lost control of their dwelling and, as a result, found comfort at workplaces or in public spaces:

*So, there was always stuff going missing from my house. Finally, I said, "I'm not buying stuff anymore. I'm not buying any more electronics. I'm not buying anything because everybody's stealing it anyway." It's just so frustrating. It just puts so much stress on me that it's almost like I view coming to work as a getaway, it's, I'm always trying to get away from my house because they've almost taken it over, you know, and it's just so stressful. Like, I can't even tell you how stressful it has been.* (54 years, father).

A sense of lack of security at home is assumed in the narrative and further evokes community insecurity. Other relatives moved out of their shared home to live in shelters or on the street because they felt their lives were threatened.

Participants who had parents with SUD also were forced to grieve the loss of their innocence when they were children. Due to the insecurity and neglect these children faced while growing up, they were placed in foster homes, where some of them were likely subjected to violence. One participant’s grandchildren were placed in foster care because of her adult child’s SUD. Not being able to intervene when the children were taken away to foster homes was a traumatic experience for her:

*The children were taken away in the middle of the night by the mobile crisis was scary even though they were being taken away due to domestic violence. They were taking care of each other, but now they got separated. I could see the fear on their face, and there is only so much comfort we can give them, for legal, to touch or to hug them* (45 years, stepdaughter).

In the above narrative, the participant was grieving the loss of these children to foster homes and the bond and caring relationship between the children, and the fear of them being separated from each other.

### Theme 2: living in dread and despair

Many participants lived with the daily fear that their loved ones would die from overdoses or chronic substance use. While most accepted that their loved one’s death from addiction-related causes was inevitable, they never felt fully prepared. One participant described the despair a father felt about his daughter’s addiction:

*Because of her [daughter], he laments, "every morning I wake up, and I'm scared to get out of bed because I don't want to find my daughter dead." He'll go into her bedroom in the mornings and stand at the foot of her bed and watch to see if he can see her breathing. Like every morning when he gets up, he goes to check to see if his daughter's still alive or if she is dead. That's how bad she is.* (44 years, sister)

The daily routine of checking on her and wondering whether she was alive or dead, as narrated above, seemed devasting enough to the participant. The fear that the addiction would cause their relative’s death was compounded by the sense of helplessness in their endeavor to intervene and halt the spiraling downward trend. The sorrow was even more poignant when relatives became addicted at a young age as it was presumed that the addiction was robbing them of productive life, noted below.

*You almost must [be prepared for that eventuality], and that's sad. He's 34 years old, and he's got a child. He's got so much to live for, but he probably can't see it, I guess. It really hurts, now, knowing Mom is gone. Myself being a parent, I can't imagine what that would feel like being your child. It hurts so bad being a sibling. Just imagine it being your child* (38 years, sister).

Participants were wearied by the constant fear, worry, and dread that accompanied caring for an individual with SUD. It was evident that most of them had reached the end of their tether, and without successful mechanisms for self-help, the participants often felt alone and on the verge of breaking:

*I was thinking to myself, "I can't live like that." And I can't help her. She doesn't want my help even though I work in the field of addiction and mental health. I've tried helping her, but she doesn't want my help, and I know I can't help her. I worry. I'm worried is she's alive, and I'm worried she has enough to ear. I worry about where she's sleeping. It was like years and years, and then I told myself, "I can't be doing that to myself 'cause I'm gonna get sick." I said, "I need to help myself."* (62 years, mother).

Many participants felt that the actions and energy their families expended were ineffective because their relatives continued to use them, leaving them gasping for hope. Due to the chronicity of addictions, many lived in a perpetual crisis.

### Theme 3: living in perpetual crisis

Caregivers experienced a state of perpetual crisis caused by a chronic lack of necessities of life, abuse, neglect, and overwhelming demand for care. Addiction was intergenerational among families in environments where substances were present. Intergeneration substance use was a complex social issue to manage by those that did not use substances:

*This intergenerational addictions and dysfunction's like a train you can't stop it, you try, everything, like I put my kids through, we went to family treatment, and they all went to individual treatment when they were teenagers so, it's like you just can't stop it* (57 years, mother).

Addiction also provided an opportunity for violence and a need to escape a toxic environment, which led to family dysfunction and disintegration. Children mostly were at significant risk of being caught in the fallout, including family breakdown, parental neglect, and diverse forms of child abuse:

*Dad was a substance user, and when I was three years old, my mom left him because of his substance use and physical abuse. She then met a man who was a substance user and a drug addict who abused me in just about every way possible from the time I was three until I was about ten* (45 years stepdaughter).

The impact of SUD on fueling family crises was an extensive experience that almost every participant shared. In addition to a child or spousal abuse, childhood exposure to substances, parents being both physically and emotionally absent, and divorce and marital separation were noted. Moreover, when more than one relative had a SUD, other members of the family were inadvertently neglected:

*You will neglect the other part of your family because you're so consumed with what that child is doing. You're wondering, "Where are they? Are they safe? Are they sleeping? Are they eating? Are they alive?" Whatever it is. You're so consumed* (57 years, mother).

Not giving up on the SUD individual meant that the care they provided became a full-time job. To the study participants, this meant taking responsibility for many aspects of their loved one’s lives, including providing financial support, assuming the parental role to their grandchildren or grown children, providing necessities for living, and meeting health care expenses, such as treatment. In most instances, the long-term assumption of this role wore down the caregiver, impacting their health in the process. The perpetual need to care for a relative with SUD also affected participants’ work-life balance. Understanding factors that shaped these experiences are vital to the development of self-care strategies.

### Theme 4: mitigating the impact of substance use in the family

Participants expressed a lack of understanding of how addiction impacts families, which influences their appreciation for their need for self-care or identification of effective ways to provide support. This lack of understanding was most profound among participants who did not have prior lived experiences with substance use. Furthermore, their lack of knowledge about addiction and its trajectory to unrealistic expectations of the willingness of the individual with the addiction to enter treatment and recovery:

*They [relatives] don't understand the trigger points. They don't understand, when she's as bad as she is, it's not a choice that she's making; it's her survival cause she's so sick. They don't understand like even, even like when I say education, there needs to be something for people who can go and they can learn about what their loved one is going through, that it's not just a matter of "oh well I'm just going to wake up, and I'm going to quit today"* (44 years, sister).

Families yearned for resources to help them make sense of their experiences and provide options for dealing with their heartache. Caregivers indicated that they could not find educational resources to increase their knowledge about addiction, its impact, and treatment. The lack of available resources suggests that healthcare providers were not well informed about the impact of addiction on caregivers, how to support families, or of resources available:

*There's no education. There's nowhere for any of the families to turn, like. There is nothing. There's nothing. There are no support groups. There's no education. There are no clinics, there's no, people like my dad, there's no "let's look at the big picture, let's have some clinic and put everything together to treat you as an elderly man with all these conditions." That the direct link is my sister. None of that exists. There's a lot of work to be done* (48 years, mother).

Although some participants were not aware of self-help groups such as Alcoholics Anonymous (AA) or Al-Anon, those who did found them to be a supportive resource that helped them cope with caregiver fatigue. In these groups, they felt comfortable sharing their pain and concerns with others who truly understood what they were going through. For some, seeking counseling from a counselor without the lived experiences of being impacted by addiction was not deemed as valuable as attending AA meetings. Moreover, Al-Anon meetings were lauded because they were devoid of stigma and judgments about addiction, and members could relate to and learn from each other:

*When I go there and dare to start talking about what was happening in my house, all these other people in the room were doing this and nodding their heads as they knew. I was like, "how do you know what's going on?" Or when they would share before I even started talking, and they would talk about what was going on, I was thinking, "were you looking in my kitchen window because you're talking? You're telling a story that I know." They knew my pain* (50 years, Sister).

Given that many participants credited Al-Anon for supporting them, and a space to engage with shared experiences, families affected by addiction must be provided the opportunity to learn about and locate Al-Anon groups. Some participants used formalized counseling both as individuals and as a family. Counselors provided mental health support and were a source of knowledge on ways to cope with caregiver fatigue:

*There was a time when I saw professional help where I was seeing a psychologist for some of the stress. I keep in contact with him every so often; give him updates on how I'm doing. He gave me a lot of tools. He helped me a lot and gave me many tools on how to deal with certain things* (57 years mother).

Formal counseling was educative and helped both children and caregivers make sense of the complexities that addiction caused. Through counseling, the affected family were able to understand the need to focus on themselves, how to deal with guilt, and how to develop healthy boundaries:

*And I'm, I'm a strong believer. I mean, I've used counseling over the years when I've had situations where I've just said, "I need an outside person to listen to this and give me some clarity because inside and talking to the wrong people is not helping," so I have sought counseling over the years. It's always been a very positive thing for me. It always allows me to recognize my vulnerabilities and trigger my thoughts and channel them in a different direction* (56 years, sister).

Seeking online information on addiction demonstrated self-determination, ambition, and self-initiation. It helped caregivers not to be constrained by the resource limitations that formalized services had:

*I have done lots of online because I like to learn anyway, so I access a lot of information online. I've talked to a couple of private rehabs and investigated resources from them where I've had some online counseling with them, so I've done some of it* (56 years, sister).

Social media was also a resource for finding useful information on how to cope with caregiver fatigue. Participants noted Facebook as a repertoire where information about addiction and self-management strategies were shared by a network of people connected to it:

*She [a friend] has a Facebook group on social media, so I thought, "I'll enter there," so I did. That's where I learned so much about what I'm going through, how I can help myself, what I need to do for self-care, all about the art of enabling, and what I was doing.* (57 years, mother.)

Caregivers affected by addiction showed resilience in seeking self-care through seeking out and attending self-help groups, counselors, and online platforms. These support services helped them make sense of their experiences and lessen the pain caused by caring for an individual with an addiction.

## Discussion

From this study, it was evident that relatives were profoundly affected by caring for individuals with SUD. A significant impact of addiction was the loss of relationships with individuals with SUD and its effects on family stability and community life. The participants’ inability to have a meaningful relationship with their relatives who were suffering from SUD was grieved as a type of social death; some relatives behaved as though the person with SUD did not exist because of the person’s inability to positively impact the family’s social dimensions [24]. Socially dead individuals have lost social identity, social connectedness, and bodily integration [24, 25]. In this study, addiction was described as adversity that stripped an individual of their personhood. Thus, addiction acquired both human and social agency and acted as a social metaphor of a life-taker, as it causes social death in families.

Declaring that someone was socially dead was an ineffective coping mechanism that caregivers employed to communicate their irreconcilable inner turmoil and their sense of resignation at the destiny that awaited their loved ones due to unabated substance use [26, 27]. Rendering an individual socially dead made the grieving process possible and signaled acceptance, despite being painful and traumatic. Yet, social death’s ambiguity could overwhelm a caregiver’s coping mechanism, impacting their mental health [28, 29].

The caregiver’s perceptions of their inability to mitigate addiction’s trajectory were expressed as helplessness and hopelessness, which appeared to be further exacerbated by their lack of knowledge of addiction. It conveyed exhaustion of the inner resources needed to mitigate distress and to engage in self-care. Although many caregivers retained the hope that recovery was possible, the painful cycle of relapse or reluctance of the individual with SUD to enter treatment eventually stripped away any thread of hope [30], and the ensuing perception of the loss of control over the lives of people with substance use, aggravated family suffering [31].

Addiction is a family can increase the relatives’ vulnerability to maltreatment and instability, leading to violence, divorce, and the inability to provide for the dependents’ needs [14]. Caregivers’ experiences of emotional burdens caused feelings of confusion, anger, frustration, anxiety, depression, abandonment, anxiety, fear, embarrassment, and guilt [14]. Stigma also creates barriers for individuals with SUD, their families, and health care providers and can diminish the ability to seek and provide resources and support [32].

Despite the evident physical and mental health impact of addiction on a family, the findings from this research revealed that healthcare providers did not recognize the stress imposed by support needed to the relative. This research identified a need for specific resources to be developed for relatives. Therefore, families may continue to live with unaddressed and unresolved health needs arising from the impact of caring for someone with SUD. Supporting self-care for families affected by addiction requires recognizing caregiver distress, which may enhance caregivers’ ability to support their loved one’s recovery [3].

Despite the limitations of addiction services focusing on families, participants exhibited resiliency and motivation to find support and resources for self-care and deal with caregiver stress. Families’ resourcefulness demonstrated their determination to find practical ways to meet theirs and their family’s emotional and social needs [32]. These strategies provided encouragement and mutual learning opportunities for self-care. Al-Anon helped families become less vulnerable to addiction’s impacts, and it gave them an environment where they could relate and learn from others who shared the same experience [33].

Families provide a source of attachment, nurturing, and association [3] to their members. Substance use and addiction are family diseases because of their impact on the family unit [34]. Families experience caregiver stress due to their support of an individual with SUD [29, 35, 36]. A lack of support is a predictor of a high caregiver burden. Interventions addressing substance use and addiction must recognize its impact on families supporting a relative with a SUD [34–37]. To provide patient-centered care, health care providers need to screen for caregiver stress. Such an intervention will expand the awareness and understanding of how health care can better optimize support for relatives in recovery.

Addiction treatment modalities need to be re-evaluated to ensure that families are fully integrated into their care model. Along with the screening, the dissemination of resources needs to be enhanced and readily available to support caregivers. Further research is required to develop evidence-based interventions focusing on family self-care modalities.

This study relied on the recollection of participants’ experiences growing up in homes where substance use was rampant. Therefore, their recollection may be limited by the fact by the passage of time. Moreover, due to logistical and operational approval difficulties, we could not recruit children and youth currently living in substance use settings. Although a sustained effort was made to recruit male participants in the study, we could only recruit four men. Therefore, the perspectives of how addiction affected men as boys are limited.

## Conclusion

In this study, we explored the experiences of relatives’ perceptions of caring for an individual with an addiction and how they engage in self-care. Our analysis of interview data gathered from 21 participants revealed that caregivers experienced a crisis caused by a lack of life necessities, which contributed to abuse, neglect, and overwhelming demand for care. For many participants, addiction robbed them of a relationship with the person using substances, which was akin to losing their relative. Several factors, including inadequate knowledge about addiction’s impact on families, and lack of resources to support family caregivers, compounded the caregivers’ psychological stress. Furthermore, the participants’ lack of knowledge about addiction and its trajectory made them have unrealistic expectations for a person with SUD’s treatment outcomes.

## Data Availability

Not applicable.

## Appendix

### Interviewer guide

1. Demographic information
  - Name, age, ethnicity, occupation?
  - How would you describe yourself?
  - Any information that is relevant to substance use and addiction?
  - What were your growing up experiences?
  - Past experiences with substance use?
2. Loved one affected by addiction
  - What type of addiction does he/she contend with?
  - History and trajectory of addiction?
  - Family involvement in addiction treatment of the loved one?
3. Impact of addiction
  - Impact of addiction on the loved one?
  - Impact of addiction on the family?
  - How has addiction in your family affected you?
    i. Socially
    ii. Financially
    iii. Physically
    iv. Psychological
    v. socially
4. Do you consider yourself in need of professional help to deal with addiction in your family?
  - If so, what type of help?
  - Have you sought professional help to deal with addiction in your family?
5. Do you have any needs that you would like to be met to help you deal with addiction in your family?
6. What resources might you need to live well and support your loved one living with addiction?
  - How would they look like?
  - What should we include?

## Abbreviations

SUD: substance use disorder
ACE: Adverse childhood experiences
AA: Alcoholics Anonymous
